# A randomized control trial of the effect of yoga on *Gunas* (personality) and Health in normal healthy volunteers

**DOI:** 10.4103/0973-6131.36785

**Published:** 2008

**Authors:** Sudheer Deshpande, H R Nagendra, Nagarathna Raghuram

**Affiliations:** Department of Yoga Research, Swami Vivekananda Yoga Anusandhana Samsthana, Bangalore, India

**Keywords:** General health, guna, Yoga

## Abstract

**Objective:**

To study the efficacy of yoga on *Guna* (yogic personality measure) and general health in normal adults.

**Methods:**

Of the 1228 persons who attended introductory lectures, 226 subjects aged 18–71 years, of both sexes, who satisfied the inclusion and exclusion criteria and who consented to participate in the study were randomly allocated into two groups. The Yoga(Y) group practised an integrated yoga module that included *asanas*, *pranayama*, meditation, notional correction and devotional sessions. The control group practised mild to moderate physical exercises (PE). Both groups had supervised practice sessions (by trained experts) for one hour daily, six days a week for eight weeks. *Guna* (yogic personality) was assessed before and after eight weeks using the self-administered Vedic Personality Inventory (VPI) which assesses *Sattva* (gentle and controlled), *Rajas* (violent and uncontrolled) and *Tamas* (dull and uncontrolled).

The general health status (total health), which includes four domains namely somatic symptoms (SS), anxiety and insomnia (AI), social dysfunction (SF) and severe depression (SP), was assessed using a General Health Questionnaire (GHQ).

**Results:**

Baseline scores for all the domains for both the groups did not differ significantly (*P* > 0.05, independent samples t test). *Sattva* showed a significant difference within the groups and the effect size was more in the Y than in the PE group. *Rajas* showed a significant decrease within and between the groups with a higher effect size in the PE group. *Tamas* showed significant reduction within the PE group only. The GHQ revealed that there was significant decrease in SS, AI, SF and SP in both Y and PE groups (Wilcoxcon Singed Rank t test). SS showed a significant difference between the groups (Mann Whitney U Test).

**Conclusions:**

There was an improvement in *Sattva* in both the Yoga and control groups with a trend of higher effect size in Yoga; *Rajas* reduced in both but significantly better in PE than in Yoga and *Tamas* reduced in PE. The general health status improved in both the Yoga and control groups.

The present age of speed and competition has increased the stresses and strains resulting in an increasing prevalence of life style-related health problems.[1] One of the increasingly popular tools to overcome this new challenge is physical activity. There is growing evidence that has established the benefits of physical exercises in preventing life style-related diseases[2] such as primary prevention of diabetes,[3] prevention of cardiac diseases through control over major risk factors such as smoking, lipids, obesity and stress,[4] better quality of life of cancer patients,[5] positive health in normal persons through better physical fitness[6] and stress reduction.[7] Yoga which is considered to be a tool for both physical and mental development of an individual is being recognized around the globe only in the last century although it has been practised in India over several centuries to promote positive health and well being. It gives solace for the restless mind and can give great relief to the sick.[8,9] It has become quite fashionable even for the common man to keep fit.[10] Some use yoga for developing memory, intelligence and creativity.[11] With its multifold advantages, yoga is becoming a part of school education.[12] Specialists use it to unfold deeper layers of consciousness in their move towards spiritual perfection.[13] With growing scientific evidence, yoga is emerging as an important health behavior-modifying practice to achieve states of health, both at physical and mental levels. Several studies have demonstrated the beneficial effects of yoga on health behavior in many life style-related somatic problems such as hypertension,[14] bronchial asthma,[15] diabetes[16] including some psychiatric conditions such as anxiety neurosis[17] and depressive illness[18] etc.

The philosophy of yoga believes that somatic problems are nothing but a manifestation of an imbalance between three *Gunas* (*Sattva*, *Rajas* and *Tamas*) that go to constitute the body-mind complex of the individual.[19] Further, in the famous scriptural text, the *Gita*; a *guna* indicates a specific behavior style. *Sattva* is symbolized by purity, wisdom, bliss, serenity, love of knowledge, spiritual excellence and other noble and sublime qualities. *Rajas* is symbolized by egoism, activity, restlessness and hankering after mundane things like wealth, power, valor and comforts. *Tamas* is related to qualities such as bias, heedlessness and inertia, perversion in taste, thought and action.[20] Ill health occurs if *Rajas* or *Tamas* become dominant and the individual gets habituated to either of these response patterns. Furthermore, the *Gita* goes on to analyze the state of mind and says that when one is dominated by these two *gunas*, the individual loses mastery over the uncontrolled, speeded-up loop of sentences of the internal dialogue, which shows up as upsurges of emotions and impulsive behavior. In an ideal state of perfect health, man has the complete freedom to use any of these three patterns (Satva, *Rajas* or *Tamas*) of responses. Hence, the degree of positive health can be measured by a tool that can grade these three patterns of behavior.[19] The tool can be used for assessment of interventions used for treatment or prevention of diseases as well as for promotion of positive health. The Vedic Personality Inventory (VPI)[21] is a valid and reliable inventory that can measure the three patterns of behavior.

While Yoga is getting popular, the relative roles of yoga and physical exercises have not been studied on *gunas* and health. Hence, the present study was designed to assess the changes in the personality and overall health status after yoga as compared to physical exercise in a randomized controlled study in normal healthy volunteers.

## METHOD

### Subjects

Of the 1228 adults who attended motivational lectures, 226 subjects consented to participate in the study and were randomly allocated to two groups of equal size. After attrition, the final sample sizes were 87 in both the yoga and control groups.

Inclusion criteria were: (a) normal healthy volunteers, (b) age 18–71 years, (c) literacy and (d) scores less than 4/5 in the General Health Questionnaire.[22]

Exclusion criteria were: (a) subjects with any ailment, (b) smoking and (c) substance abuse.

Source of subjects: Normal adults were recruited from five different locations in Bangalore after public talks at different institutions such as colleges, health clubs, Rotary Clubs, Lion's clubs and big apartment complexes.

Informed consent was obtained from all the subjects who participated in the project and also from the institutional heads where the classes were conducted. The institutional ethical committee of SVYASA cleared the project proposal.

### Design

This is a prospective, randomized, single-blind, controlled study aiming to compare the efficacy of yoga (Y) and physical exercise (PE) in normal healthy volunteers in a South Indian population. Introductory lectures were arranged in public centers such as colleges, health clubs, Rotary clubs, Lion's clubs and apartment complexes. The classes were planned in five different centers in the city of Bangalore. Two hundred and twenty-six persons who consented to participate in the study and satisfied the inclusion and exclusion criteria were randomly allotted to two groups by using five random number tables (different table for each center) generated from the random number generator program.[23] The experimental group was given Y practices and the control group was given PE for one hour daily on empty stomach (6 to 7 a.m.). The classes were conducted six days a week for eight weeks and attendance was maintained by the teachers. Trained experts (in yoga for the Y group and PT for the PE group) conducted parallel sessions for the two groups in different rooms in the same venue. It was ensured that there was no interaction between the subjects. The tests were self-administered before and eight weeks after the intervention. Arrangements were made for the subjects to sit in a quiet place free from distractions and influence from other people.

Masking: The answered questionnaires were coded and kept away for future scoring. A psychologist who was not involved in the subject allocation or supervision of the classes scored the questionnaires which were decoded only after the scoring of both the before and after data was completed.

### Assessments

Assessments were done using the following questionnaires:

1. The Vedic Personality Inventory (VPI): In 1998, Wolf developed an inventory to assess three personality constructs (*gunas*) based on their description in the most ancient Indian scriptures called Vedas. Hence, this inventory was named the VPI and it measures the three *gunas*—*Sattva*, *Rajas* and *Tamas*. It has 30 items for the *Sattva* guna, 28 for rajoguna and 32 for tamo guna. VPI has good internal consistency and reliability with Cronbach's alpha ranging from 0.850 for *Sattva*, 0.915 for *Rajas* and 0.699 for *Tamas*. In terms of discriminant validity, all but one facet had significant differences.[21]
2. General Health Questionnaire (GHQ): The GHQ designed by Goldberg in order to identify psychiatric morbidity in general practice, is a self-administered questionnaire (English version). It has 28 items with four subscales to measure somatic symptoms (SS), anxiety and insomnia (AI), social dysfunction (SF) and severe depression (SP). It provides information about the recent mental status, thus identifying the presence of possible psychiatric disturbance. This questionnaire has acceptable psychometric properties and has good internal consistency and reliability with Cronbach's alpha of 0.85 and validity of 0.76.[24]

## INTERVENTION

### Yoga group

The Integrated yoga module was selected from the integrated set of yoga practices used in earlier studies on the effects of yoga for positive health.[25] This integrated approach is developed based on ancient Yoga texts[26] to bring about a total development at physical, mental, emotional, social and spiritual levels.[27] The techniques include physical practices (*kriyas*, *asanas*, a healthy yoga diet), breathing practices with body movements and Pranayama, meditation, devotional sessions, lectures on yoga, stress management and lifestyle change through notional corrections for blissful awareness under all circumstances (action in relaxation). Yoga was taught by qualified yoga teachers.

### Physical exercise group

The set of physical exercises were standard execises[28] meant to provide mild to moderate activity designed by experts in physical education.

### Data extraction

The scoring of the questionnaires was carried out as per the instructions in the manuals. The structure of these questionnaires is described below:

1. VPI evaluates the *Sattva*, *Rajas* and *Tamas gunas* by using a 7-point Likert-type scale. Scores for the *gunas* are obtained by adding the responses for the items for a guna and then dividing by the number of items for that mode. For each subscale, a higher score indicates a greater predominance of that mode. The minimum and maximum possible scores for the three domains range from 1–7.
2. GHQ: This 28 item test using a binary method of scoring (0, 0, 1, 1) yields an assessment on four robust subscales: somatic symptoms (SS), anxiety and insomnia (AI), social dysfunction (SF) and severe depression (SP). A sum of the scores for these four subscales gives the score for total health. The lower the scores in the GHQ, the better the state of health. The cut-off scores for the GHQ used for this study were 4 or 5 (4/5).[22]

### Statistical analysis

Data was analyzed using the SPSS package version 10.0.

Based on a previous study,[29] the effect size was calculated to be 0.8. With a power of 0.8 and alpha set to 0.05, the minimum sample size was found to be 164. This calculation was done using G power.[30] The size of the sample actually used was 174.

Data at baseline was assessed for normal distribution using Shapiro-Wilk's test for both the groups. Independent samples t-test was done for checking homogeneity of baseline scores of the two groups. Paired samples t test and independent samples t test were used for VPI which had normally distributed data and Wilcoxon's signed ranks and Mann Whitney U tests were used for GHQ data which were not normally distributed. An independent samples t test was done to analyze between the groups and paired samples test within groups. The effect size of the study (mean A – mean B)/ standard deviation (SD) of difference scores) is an absolute measure of the difference that exists between the populations for a parameter, a concept first introduced by the sociologist, J. Cohen.[31]

As the study population had a wide age range, statistical analysis was also carried out by grouping them as juniors (age ≤ 24 years) and seniors (age > 24 years) based on the median age. The independent samples t-test for between groups and paired samples t test for within groups were conducted for the two age groups. The data was also analyzed using gender as a factor.

## RESULTS

Figure 1 shows the study profile wherein of 1228 subjects who attended the motivational lectures, only 226 who satisfied the inclusion and exclusion criteria were selected and randomly allotted to the Y and PE groups. The reasons for dropout of 52 subjects are shown in Figure 1.

**Figure:**
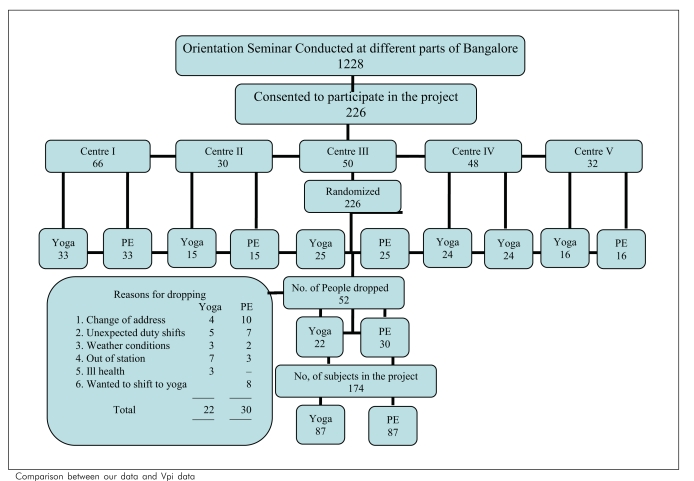
Comparison between our data and Vpi data

Table 1 shows the demographic data. There were 87 subjects (40 females) in each group aged 18–71 years, the mean age being 29.44 ± 11.94 years. They belonged to different callings such as college students, professionals, housewives and retired persons.

**Table 1:** Demographic data for VPI

Age	Sex	Y (*n* = 87) 31.33±11.9 5	PE (*n* = 87) 32.35±11.32
≤ 24 years (Juniors)	Male (m±SD)	26.79±12.20	28.00±11.76
	Female (m±SD)	20.00±1.75	20.29±1.44
> 24 years (Seniors)	Male (m±SD)	20.61±1.82	20.73±1.89
	Female (m±SD)	38.88±9.55	30.85±8.56
Gender	Male (m±SD)	41.36±13.89	40.82±10.85
	Range	18–71	18–58
	Female	40	40
Categories	Male	47	47
	Students	49	44
	Employees	18	30
	Housewives	10	7
	Business	10	6

The baseline values were normally distributed for *Tamas* (*P* = 0.209) and *Sattva* (*P* = 0.717) and were well-matched for all three domains (Independent samples t-test).

Table 2 shows the comparison of the baseline scores for the three *gunas* of the VPI with the norms provided in the manual. It showed that the scores are within the predicted normal range. The mean value is marginally higher for *Sattva* and lower for *Rajas* and *Tamas* in the South Indian population selected in the present study as compared to the norms from studies in the USA.

**Table 2:** VPI scores for yoga and control groups—comparison of means (paired samples test)

	Before Means±SD Y	After Means±SD Y	*P* value	Effect Size	Before Means±SD PE	After Means±SD PE	*P* value	Effect Size
*Tamas*	3.12 ± 0.51	2.97 ± 0.91	0.095	0.18	3.24 ± 0.67	2.99 ± 0.69	0.001	0.36
*Rajas*	3.83 ± 0.62	3.72 ± 0.51	0.12	0.17	3.67 ± 0.62	3.43 ± 0.79	0.002*	0.33
*Sattva*	4.88 ± 0.52	5.26 ± 0.51	<0.001	0.61	4.91 ± 0.53	5.21 ± 0.65	<0.001	0.45

* *Rajas* showed a significant difference between the groups (*P* = 0.005) (Independent Samples Test); (Effect size = difference in means (after–before)/SD of the difference scores)

**Table 3:** VPI scores in age groups - Age ≤ 24 years and > 24 years (paired-samples t test)

		Before Means±SD Y	After Means±SD Y	*P* value	Before Means±SD PE	After Means±SD PE	*P* value
Age ≤ 24 years	*Tamas*	3.16 ± 0.49	3.20 ± 1.63	0.774	3.28 ± 0.67	3.16 ± 2.13	0.4
	*Rajas*	3.84 ± 0.66	3.99 ± 0.74	0.286	3.75 ± 0.63	3.56 ± 0.75	0.152
	*Sattva*	4.67 ± 0.47	5.26 ± 0.55	<0.001	4.79 ± 0.44	5.14 ± 0.65	0.002
Age > 24 years	*Tamas*	3.09 ± 0.53	2.67 ± 0.69	0.001	3.21 ± 0.68	2.83 ± 0.77	0.001
	*Rajas*	3.81 ± 0.61	3.51 ± 0.57	0.002	3.62 ± 0.62	3.31 ± 0.83	0.015
	*Sattva*	4.91 ± 0.59	5.12 ± 0.45	0.001	5.00 ± 0.59	5.09 ± 0.62	0.014

**Table 4:** Gender-based VPI scores (paired samples t test)

		Before Means±SD Y	After Means±SD Y	*P* value	Before Means±SD PE	After Means±SD PE	*P* value
Females	*Tamas*	3.15 ± 0.52	2.80 ± 1.04	0.04	3.20 ± 0.71	2.97 ± 0.71	0.053
	*Rajas*	3.66 ± 0.62	3.43 ± 0.48	0.502	3.64 ± 0.63	3.50 ± 0.80	0.196
	*Sattva*	4.91 ± 0.42	5.20 ± 0.50	0.004	4.98 ± 0.58	5.23 ± 0.62	0.034
Males	*Tamas*	3.11 ± 0.50	3.10 ± 0.58	0.924	3.28 ± 0.65	3.01 ± 0.46	0.032
	*Rajas*	3.96 ± 0.63	3.96 ± 0.41	0.898	3.73 ± 0.63	3.50 ± 0.79	0.014
	*Sattva*	4.86 ± 0.60	5.33 ± 0.52	<0.001	4.80 ± 0.49	5.19 ± 0.68	0.001

***Tamas*:** The PE group showed a significant decrease in the *Tamas* score from 3.24 to 2.99 (*P* = 0.001) (paired samples t test). The senior subjects (age > 24 years) in both the Y (3.09 to 2.67) and PE (3.21 to 2.83) groups showed a significant decrease (*P* = 0.001). In gender analysis, females showed a decrease with Y (*P* = 0.040) and males showed a decrease with PE (*P* = 0.032).

***Rajas*:** The PE group showed a significant decrease in scores from 3.67 to 3.43 (*P* = 0.002). Seniors in both the Y (3.81 to 3.51) (*P* = 0.002) and PE (3.62 to 3.31) groups (*P* = 0.015) have shown significant decreases. In gender analysis, males showed a decrease with PE (3.73 to 3.37) (*P* = 0.014). Significantly greater reduction was observed in the PE than in the Y group (*P* = 0.005) and in juniors (*P* = 0.012).

***Sattva*:** *Sattva* scores have increased significantly in both Y (4.88 to 5.26) (*P* = 0.001) and PE (4.91 to 5.21) (*P* < 0.001) groups with a greater effect size in the Y (0.61) than in the PE (0.45) group. Juniors, seniors, males and females in both the Y and PE groups have all shown significant increase in *Sattva* scores.

Table 5 shows the results for all variables of the GHQ.

**Table 5:** GHQ scores (Wilcoxon signed ranks test)

	Before Means±SD Y	After Means±SD Y	*P* value	Before Means±SD PE	After Means±SD PE	*P* value
SS	0.57 ± 0.91	0.29 ± 0.65	<0.001	0.41 ± 0.80	0.11 ± 0.32	0.001
AI	0.61 ± 0.92	0.08 ± 0.38	<0.001	0.49 ± 0.90	0.18 ± 0.74	0.011
SF	0.60 ± 0.91	0.15 ± 0.39	<0.001	0.60 ± 0.99	0.23 ± 0.52	0.001
SP	0.44 ± 0.73	0.22 ± 0.58	0.017	0.52 ± 0.65	0.15 ± 0.42	<0.001
TH	2.22 ± 2.48	0.74 ± 1.21	<0.001	2.02 ± 2.78	0.68 ± 1.28	<0.001

SS: Somatic symptoms; AI: Anxiety and insomnia; SF: Social dysfunction; SP: Severe depression; TH: Total health

**Table 6:** GHQ scores: Age ≤ 24 years and > 24 years (Wilcoxon signed ranks test)

		Before Means±SD Y	After Means±SD Y	*P* value	Before Means±SD PE	After Means±SD PE	*P* value
Age ≤ 24 years	SS	0.65 ± 0.93	0.43 ± 0.76	0.161	0.43 ± 0.76	0.14 ± 0.35	0.01
	AI	0.71 ± 0.96	0.10 ± 0.47	<0.001	0.66 ± 0.99	0.30 ± 1.00	0.057
	SF	0.80 ± 0.98	0.18 ± 0.44	<0.001	0.75 ± 1012	0.34 ± 0.64	0.019
	SP	0.45 ± 0.71	0.29 ± 0.68	0.185	0.64 ± 0.89	0.16 ± 0.43	<0.001
	TH	2.61 ± 2.54	1.00 ± 1.44	<0.001	2.48 ± 3.11	0.93 ± 1.53	0.001
Age > 24 years	SS	0.47 ± 0.89	0.11 ± 0.39	0.004	0.40 ± 0.85	0.09 ± 0.29	0.044
	AI	0.47 ± 0.86	0.05 ± 0.23	0.002	0.33 ± 0.78	0.06 ± 0.26	0.047
	SF	0.34 ± 0.75	0.11 ± 0.31	0.071	0.44 ± 0.83	0.12 ± 0.32	0.017
	SP	0.42 ± 0.76	0.13 ± 0.41	0.047	0.40 ± 0.79	0.14 ± 0.41	0.013
	TH	1.71 ± 2.25	0.39 ± 1.00	0.001	1.56 ± 2.00	0.42 ± 0.00	0.003

SS: Somatic symptoms; AI: Anxiety and insomnia; SF: Social dysfunction; SP: Severe depression; TH: Total health

**Table 7:** Gender-based GHQ scores (Wilcoxon signed ranks test)

		Before Means±SD Y	After Means±SD Y	*P* value	Before Means±SD PE	After Means±SD PE	*P* value
Females	SS	0.50 ± 0.99	0.25 ± 0.58	0.115	0.40 ± 0.74	0.07± 0.27	0.018
	AI	0.50 ± 0.85	0.02± 0.16	0.001	0.57 ± 0.98	0.30 ± 1.04	0.208
	SF	0.40 ± 0.81	0.10 ± 0.30	0.038	0.45 ± 0.81	0.15 ± 0.36	0.038
	SP	0.35 ± 0.62	0.28 ± 0.72	0.584	0.50 ± 0.85	0.10 ± 0.45	0.005
	TH	1.71 ± 2.35	0.65 ± 1.03	0.01	1.93 ± 2.80	0.70 ± 1.44	0.018
Males	SS	0.64 ± 0.85	0.32 ± 0.69	0.027	0.43 ± 0.85	0.15± 0.36	0.022
	AI	0.70 ± 0.98	0.13± 0.49	<0.001	0.43 ± 0.83	0.08± 0.28	0.007
	SF	0.77 ± 0.96	0.19 ± 0.45	<0.001	0.72 ± 1.12	0.30 ± 0.62	0.009
	SP	0.51 ± 0.80	0.17 ± 0.43	0.008	0.53 ± 0.86	0.13 ± 0.40	<0.001
	TH	2.62 ± 2.53	0.81 ± 1.36	<0.001	2.11 ± 2.78	0.66 ± 1.15	<0.001

SS: Somatic symptoms; AI: Anxiety and insomnia; SF: Social dysfunction; SP: Severe depression; TH: Total health

**TABLE 8:** Comparison between our data (before and after) and standard VPI data

	*n*	Observed range	Observed mean±SD	*n*	Predicted range	Predicted mean±SD
*Sattva*		3.04 - 6.17	4.90±0.53		3.00 - 6.39	4.67±0.75
*Rajas*	174	2.11 - 5.25	3.76±0.63	247	2.46 - 5.96	4.07±1.08
Tamas		1.47 - 5.38	3.19±0.60		1.43 - 6.00	3.49±0.90

**Somatic symptoms (SS):** SS symptoms have reduced significantly in both Y (0.57 to 0.29) (*P* = 0.011) and PE (0.41 to 0.11) (*P* = 0.001) groups. Juniors, seniors, males and females of the PE group have shown significant decrease in SS. Seniors and males in the Y group have shown significant decrease in SS. There was a significant difference between the groups.

**Anxiety and insomnia (AI):** AI symptoms have decreased significantly in both the Y (0.61 to 0.08) (*P* < 0.01) and PE (0.49 to 0.18) (*P* = 0.011) groups. Juniors, seniors, females and males in the in Y group have shown significant decrease in AI whereas only seniors and males have shown significant decrease in AI in the PE group.

**Social dysfunction (SF):** A significant decrease was observed in both the Y (0.60 to 0.15) (*P* ≤ 0.001) and PE (0.60 to 0.23) (*P* = 0.001) groups. Juniors, females and males have shown significant decrease in SD with Yoga whereas juniors, seniors, males and females have shown significant decrease in SD due to PE.

**Severe depression (SP):** Both Y (0.44 to 0.22) (*P* = 0.017) and PE (0.52 to 0.15) (*P* < 0.01) groups have shown significant reduction in SP. Juniors, seniors, females and males have shown a significant decrease in SP due to PE. Only seniors and males have shown a significant decrease in SP due to yoga.

## DISCUSSION

This is a randomized, controlled, prospective study in normal adults comparing the efficacy of yoga with a control intervention of PE of eight weeks in 174 normal adults on changes in their personality (*guna*) and General health as assessed by VPI and GHQ. The results showed that there was an increase in *Sattva scores* (*P* < 0.001) in both Y and PE groups and a decrease in *Rajas* (*P* = 0.002) and *tamas* (*P* = 0.01) scores in the PE group. The scores for *Tamas* decreased significantly in seniors of both the groups (females in Y and males in PE) (paired samples t test). The increase in *Sattva* scores was higher in the Y group (effect size 0.61) than in the PE group (effect size 0.45) (paired samples t test). The decrease in the *Rajas* scores was significantly higher in the PE than in the Y (*P* =0.005) (independent samples t-test) groups and this was seen in juniors and males. The GHQ revealed a significant improvement on all four domains and the overall health in both groups after the intervention (*P* ≤ 0.001) (Wilcoxon's signed rank test). It can be seen from the GHQ scores that PE was more effective in reducing somatic symptoms (*P* = 0.018) (Mann Whitney test), severe depression (effect size for Y = 1.46, PE = 1.60) and anxiety and insomnia (effect size for Y = 0.98, PE = 1.93).

A similar study by Dasa[32] conducted by the use of *mahamantra* in a three-armed, randomized prospective, controlled study on 62 volunteers showed that the *mahamantra* group had increased *Sattva* and decreased *Tamas* with no significant change in *Rajas* scores on the VPI questionnaire after a month of chanting of *mahamantra*, 20 minutes daily for four weeks. In the present study, apart from an increase in *Sattva* and decrease in *Tamas*, there is a significant decrease in *Rajas* which was not observed after *Mahamantra*. This difference could be because of the inclusion of *Asanas* and *Pranayama* to the Meditation technique in the integrated yoga program used in the present study as compared to the *mahamantra* which is mainly a form of meditation. In their study, Dasa *et al.* also showed a significant reduction in stress, anxiety and depression after *mahamantra* as measured by State Trait Anxiety Inventory (STAI) comparable to the results of GHQ in this study.

The behavior of a human being is an expression of a combination of different *gunas*. *Tamas* (meaning darkness) is the grossest aspect of our personality characterized by excessive sleep, innocence, laziness, depression, procrastination, a feeling of helplessness, impulsivity, anger and arrogance (packed up with vital energy). When we reduce *Tamas* through mastery over the mind, we become dynamic, sensitive and sharp to move towards *Rajas* (the shining one) characterized by intense activity, ambitiousness, competitiveness, high sense of self importance, desire for sense gratification, little interest in spiritual elevation, dissatisfaction with one's position, envy of others and a materialistic cleverness^.^[33] With further growth and mastery, one moves into *Sattva* -a dominance which includes the qualities of truthfulness, stability, discipline, sense of control, sharp intelligence, preference for vegetarianism, truthfulness, gravity, dutifulness, detachment, respect for superiors and staunch determination[21] and stability in the face of adversity and also conscious action. Thus, we can see that although both *Rajas* and *Tamas* have both positive and negative qualities, they are the manifestation of a violent state of mind in which a person lacks mastery over upsurges of emotions and impulsive behaviour.[33] Most of the qualities of *Sattva* which are manifestation of a calm state of mind are achievable by different yoga techniques (physical postures, *pranayama* and/or meditation) meant for mastery over the mind-body complex.[34] Several earlier studies have independently corroborated these notions. It has been shown that self esteem as well as the sense of control and determination improved after meditation.[35] Reduction in crime rate after transcendental meditation (TM) supported the effect of a calm state of mind on social health.[36] These positive effects also show up as better perception and memory as well as better motor performance (dexterity and coordination tests).[37] Better academic performance has also been documented.[38]

Although in this study, Yoga has shown a better effect on the *Sattva* guna than PE with a better effect size, the main difference between Y and PE practices seems to be the effect on *rajas guna*. The reduction in this guna was significantly higher after PE than after Y (this group difference was in males and juniors). The scores for *Tamas* also decreased significantly in seniors of both groups (females in Y and males in PE groups) with the effect size being higher in the PE than in the Y groups. Thus, significantly greater reductions in *Rajas* and *Tamas* were worthy of note with PE than with Y. This positive effect of PE in reducing *Rajas* and *Tamas* adds to the fund of knowledge about several psycho-physiological benefits of PE. Hence, it appears that physical practices are more effective in reducing the limitations of *Rajas* and *Tamas* such as lack of mastery over upsurges of emotions and impulsive behavior, while yoga improves the softer qualities of *Sattva*. The mechanism of how physical exercises may reduce *Rajas* and *tamas* and how yoga may increase *Sattva* needs to be investigated by further studies. Thus, we may conclude that both physical activity (to reduce *Rajas* and *Tamas*) and Yoga (to improve *Sattva*) may be recommended for the harmonious promotion of personality.

The GHQ showed significant differences within groups in all domains in both groups. There was a significant difference in SS between the Y and PE groups (Mann Whitney Test).

Observations by Atlantis *et al.* on the efficacy of physical exercise practised for eight weeks in a population of Australian employees showed that the intervention significantly improved the Quality of Life as compared to a waiting list control group (measured by SF-36). They have shown an improvement of 12.8% in physical functioning, 9.90% in general health, 44.50% in vitality and 15.90% in mental health scores.[29] The significantly better reduction in SS in the Yoga group in our study may be due to deeper rest and relaxation obtained in Yoga.

The results of the study seem to point out clear differences between Y and PE on VPI whereas differences between Y and PE are not found in most domains of GHQ (except SS). Hence, although GHQ is a good measure of the various aspects of health and disease, VPI seems to be a better measure to differentiate the effects of Y and PE.

In summary, this randomized, prospective, single-blind, comparative study has shown the efficacy of both Y and PE in improving all components of general health. While physical exercise has reduced *Rajas* and *Tamas*, the yogic practice has increased *Sattva*. Hence, yoga which is more traditionally practised in India and cost-effective, can be recommended with additional benefits of promotion of the *Sattva guna*.

The strength of our design is a PE intervention matched with the integrated Y module. The study population was taken from different parts of Bangalore from different socioeconomic classes of the city. The improvement observed in both groups after eight weeks of intervention in all variables in both groups not only provides hitherto undemonstrated evidence of the efficacy of physical activity in a normal South Indian adult population but also shows that yoga could be an equally effective tool. This study also brings out the subtle differences in the efficacy of the two interventions (Y or PE). It also points out the utility of the VPI as a tool for measuring the subtle dimensions of *guna* described in traditional texts of yoga that can measure the steps of growth of an individual.

## References

[1] Dhirendra B (1968). Yoga for life and living.

[2] Margareta Eriksson K, Westborg CJ, Eliasson MC (2006). A randomized trial of lifestyle intervention in primary healthcare for the modification of cardiovascular risk factors. Scand J Public Health.

[3] Brukner PD, Brown WJ (2005). Is exercise good for you?. Med J Aust.

[4] Stampfer M, Hu F, Manson J, Rimm E, Willett W (2000). Primary prevention of coronary heart disease in women through diet and lifestyle. N Engl J Med.

[5] Courneya KS, Friedenreich CM (1999). Physical exercise and quality of life following cancer diagnosis: A literature review. Ann Behav Med.

[6] Lamb KL, Brodie DA, Roberts K (1988). Physical fitness and health-related fitness as indicators of a positive health state. Health Promotion Int.

[7] Dimeo F, Bauer M, Varahram I, Proest G, Halter U (2001). Benefits from aerobic exercise in patients with major depression: A pilot study. Br J Sports Med.

[8] Bloomfield HH, Cain MP, Jaffe DT (1975). ‘TM’-Discovering inner Energy and overcoming stress.

[9] Brena SH (1975). Yoga and Medicine.

[10] Pratinidhi BP (1966). The ten point way to health.

[11] Denniston D, Williams PM (1975). ‘TM’ book.

[12] Sarasvati, Swami (1975). Yoga for vital beauty.

[13] Nirmala, G (1978). Report No. KK/20.

[14] McCaffrey R, Ruknui P, Hatthakit U, Kasetsomboon P (2005). The effects of yoga on hypertensive persons in Thailand. Holist Nurs Pract.

[15] Sabina AB, Williams AL, Wall HK, Bansal S, Chupp G, Katz DL (2005). Yoga intervention for adults with mild-to-moderate asthma: A pilot study. Ann Allergy Asthma Immunol.

[16] Bijlani RL, Vempati RP, Yadav RK, Ray RB, Gupta V, Sharma R (2005). A brief but comprehensive lifestyle education program based on yoga reduces risk factors for cardiovascular disease and diabetes mellitus. J Altern Complement Med.

[17] Brown RP, Gerbarg PL (2005). Sudarshan Kriya yogic breathing in the treatment of stress, anxiety and depression: Part I-neurophysiologic model. J Altern Complement Med.

[18] Jorm AF, Christensen H, Griffiths KM, Rodgers B (2002). Effectiveness of complementary and self-help treatments for depression. Med J Aust.

[19] Goyandka J (1999). Srimadbhagavad *gita* Tattvavivecani.

[20] Das RC (1991). Standardization of the *Gita* inventory of personality. J Indian Psychol.

[21] Wolf DB (1998). The vedic personality inventory: A study of the *Gunas*. J Indian Psychol.

[22] Goldberg DP, Gater R, Sartorius, Ustan TB, Piccinelli M, Gujeje O (1997). The validity of two versions of the GHQ in the WHO study of mental illness in general health care. Psychol Med.

[23] http://www.randomisor.org.

[24] Goldberg DP, Hillier VF (1979). A scaled version of the general Health Questionnaire. Psychol Med.

[25] Nagarathna R, Nagendra HR (2003). Integrated Approach of Yoga Therapy for Positive Health.

[26] Lokeswarananda S, Taittiriya U (1996).

[27] Nagarathna R, Nagendra HR (2003). Yoga.

[28] Nagarathna R, Nagendra HR (2001). Yoga for Arthritis.

[29] Atlantis E, Chow CM, Kirby A, Singh MF (2004). An effective exercise-based intervention for improving mental health and quality of life measures: A randomized controlled trial. Prev Med.

[30] http://www.uni-mannhein.de/gpower.

[31] Cohen J (1977). Statistical power analysis for the behavioral sciences.

[32] Dasa DG (1999). Effects of the Hare Krsna Maha mantra on stress, Depression and The Three *Gunas*. VNN Vaishnava News org Networh VNN4267.

[33] Nagendra HR (2003). The secret of action.

[34] Holt WR, Caruso JL, Riley JB (1978). Transcendental Meditation vs pseudo-meditation on visual choice reaction time. Percept Motor Skills.

[35] Alexander CN, Robinson P, Rainforth M (1994). Treating and preventing alcohol, nicotine and drug abuse through transcendental meditation: A review and statistical meta-analysis. Alcoholism Treatment Quarterly.

[36] Abrams AI (1979). Transcendental meditation and rehabilitation at Folsom prison: Response to a critique. Criminal Justice Behav.

[37] Dillbeck MC, Orme-Johnson DW (1987). Physiological differences between transcendental meditation and rest. Am Psychol.

[38] Kember P (1985). The Transcendental Meditation technique and postgraduate academic performance. Br J Educ Psychol.

